# Combination of ATO with FLT3 TKIs eliminates FLT3/ITD+ leukemia cells through reduced expression of FLT3

**DOI:** 10.18632/oncotarget.25972

**Published:** 2018-08-31

**Authors:** Kozo Nagai, Lihong Hou, Li Li, Bao Nguyen, Tessa Seale, Courtney Shirley, Hayley Ma, Mark Levis, Gabriel Ghiaur, Amy Duffield, Donald Small

**Affiliations:** ^1^ Department of Oncology, Johns Hopkins University School of Medicine, Baltimore, MD, USA; ^2^ Department of Pathology, Johns Hopkins University School of Medicine, Baltimore, MD, USA; ^3^ Department of Pediatrics, Johns Hopkins University School of Medicine, Baltimore, MD, USA

**Keywords:** acute myeloid leukemia, FLT3, internal tandem duplication, tyrosine kinase inhibitor, arsenic trioxide

## Abstract

Acute myeloid leukemia (AML) patients with FLT3/ITD mutations have a poor prognosis. Monotherapy with selective FLT3 tyrosine kinase inhibitors (TKIs) have shown transient and limited efficacy due to the development of resistance. Arsenic trioxide (ATO, As_2_O_3_) has been proven effective in treating acute promyelocytic leukemia (APL) and has shown activity in some cases of refractory and relapsed AML and other hematologic malignances. We explored the feasibility of combining FLT3 TKIs with ATO in the treatment of FLT3/ITD+ leukemias. The combination of FLT3 TKIs with ATO showed synergistic effects in reducing proliferation, viability and colony forming ability, and increased apoptosis in FLT3/ITD+ cells and primary patient samples. In contrast, no cooperativity was observed against wild-type FLT3 leukemia cells. ATO reduced expression of FLT3 RNA and its upstream transcriptional regulators (HOXA9, MEIS1), and induced poly-ubiquitination and degradation of the FLT3 protein, partly through reducing its binding with USP10. ATO also synergizes with FLT3 TKIs to inactivate FLT3 autophosphorylation and phosphorylation of its downstream signaling targets, including STAT5, AKT and ERK. Furthermore, ATO combined with sorafenib, a FLT3 TKI, *in vivo* reduced growth of FLT3/ITD+ leukemia cells in NSG recipients. In conclusion, these results suggest that ATO is a potential candidate to study in clinical trials in combination with FLT3 TKIs to improve the treatment of FLT3/ITD+ leukemia.

## INTRODUCTION

Over the last few decades, the outcome of patients with AML has been improved by development of intensive chemotherapy, risk-based stratification, and stem cell transplantation [1]. However, overall long-term survival remains poor largely due to refractory and relapsed disease. Internal tandem duplication (ITD) mutations in the juxtamembrane domain (JM) of FLT3 are one of the most frequently occurring mutations (~25%) in AML and are associated with an increased relapse risk and decreased disease-free survival [2, 3]. In an attempt to improve the cure rate for these AML patients, several potent FLT3 TKIs have been developed. However, efficacy of monotherapy with FLT3 TKIs has been limited as a result of a variety of resistance mechanisms [4–8]. Combinatorial strategies will be clinically important to reverse resistance and improve outcome in FLT3/ITD+ AMLs.

Arsenic trioxide (ATO, As_2_O_3_) is an inorganic compound which has been proven successful in treating PML-RARα positive acute promyelocytic leukemia (APL) through SUMOylation and ubiquitination of the PML-RARα mutant [9–12]. Other than PML-RARα, ATO has been reported to cause proteasome-dependent degradation of mutant p53, cytoplasmic NPM1 mutation (NPMc) and BCR-ABL oncoprotein, resulting in apoptosis of leukemia cells expressing these mutations [13–17]. In addition, ATO also affects a variety of other targets including JNK, NF-κB, thioredoxin reductase, and MAPK, and is involved in the reactive oxygen species (ROS) production [18–22]. These mechanisms enable it to exert anti-tumor effects against a wide range of cancers, including non-APL AML, non-AML hematological malignancies and solid tumors [23, 24]. Therefore, ATO has also been tested in a number of clinical trials for the treatment of non-APL AML with the demonstration of some clinical activity. However, as compared to APL, non-APL myeloid leukemia cells are less sensitive to the pro-apoptotic effects of ATO [25–27]. The clinical application of ATO has been somewhat limited by its toxicity to heart, liver, kidney and the nervous system, especially the cardiac toxicity caused by high concentrations of ATO [28]. This has led to a search for approaches to combine ATO with chemotherapy or other agents.

Recently, several studies have reported the synergistic effects of ATO and FLT3 TKIs on FLT3 mutant leukemia cells [29–31]. Although several signaling pathways, including those involving STAT5, ERK, AKT, GSK3β are mentioned to be affected by the combination of the 2 drugs, the underlying mechanisms have not been fully illustrated. Here we investigate the feasibility of combining ATO with FLT3 TKIs, with a specific focus on understanding ATO's effect on the degradation of mutant FLT3 protein, as a strategy to increase the efficacy of FLT3 TKIs in the aim to improve the treatment of FLT3/ITD+ leukemia.

## RESULTS

### ATO synergizes with FLT3 TKIs to inhibit the proliferation and colony formation of FLT3 mutant leukemic cells

**Figure 1:**
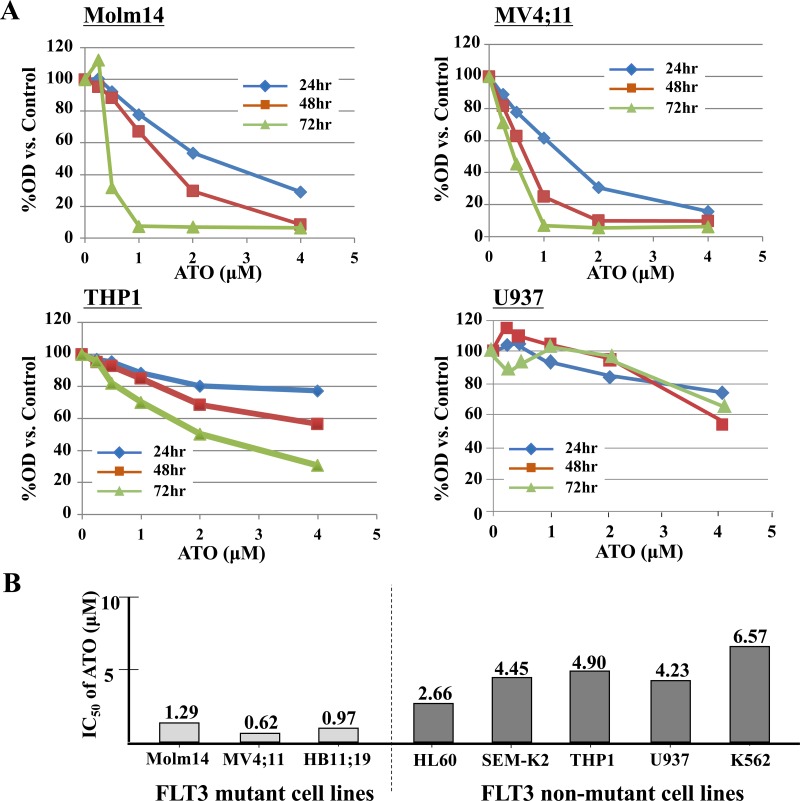
ATO has relatively selective anti-proliferative effects against FLT3/ITD + leukemia cells **A.** FLT3/ITD+ (Molm14, MV4;11), FLT3/WT (THP1) or FLT3 negative cells (U937) were treated with ATO for up to 72 hours. Cells were subjected to MTT-based cell proliferation assays. **B.** IC_50_ was calculated based on the MTT assay results at 48 hours.

We first tested the anti-proliferative effect of ATO against a variety of non-APL leukemic cells. Proliferation assay demonstrated that FLT3 mutant cells (Molm14, MV4;11 and HB11;19) were more sensitive to ATO compared with FLT3/WT cells (SEMK-2 and THP1) or FLT3-negative cells (HL60, K562, and U937) (Figure 1A and Supplementary Figure 1). The IC_50_ of ATO was significantly lower against the FLT3-mutant cells compared to the FLT3/WT and FLT3-negative cells (Figure 1B). This suggests that ATO may exert a stronger effect on FLT3 mutant leukemic cells.

**Figure 2:**
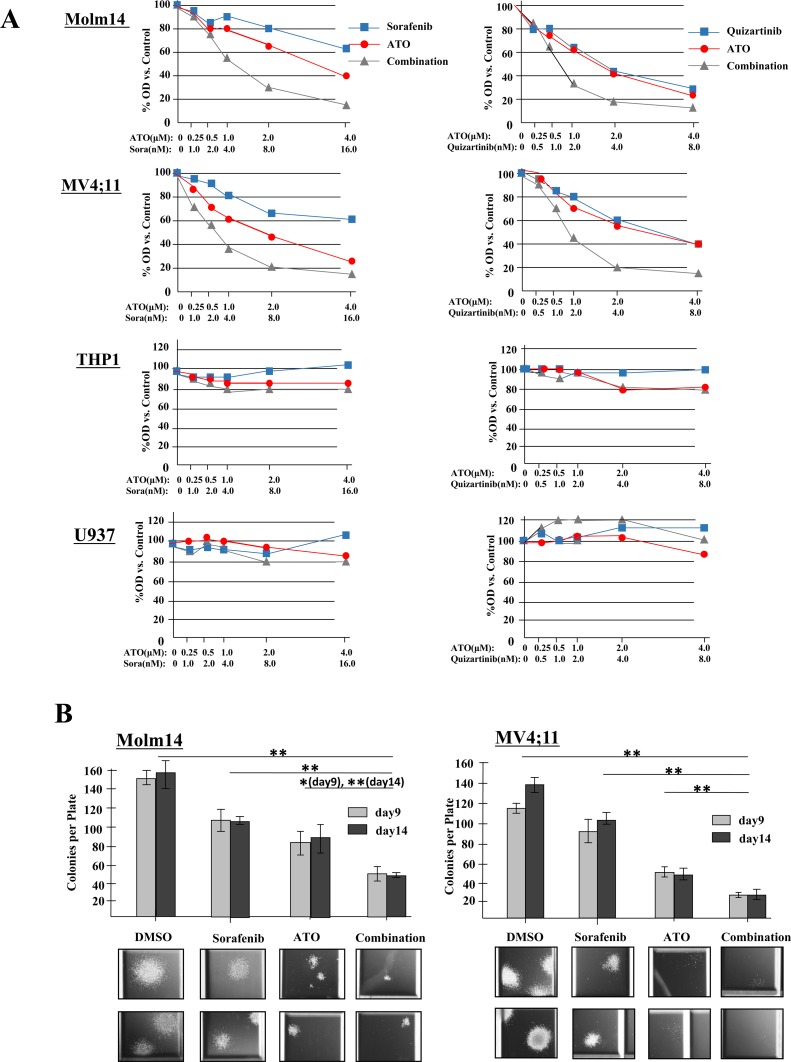
ATO synergizes with FLT3 TKIs to reduce proliferation and clonogenicity of FLT3/ITD+ cell lines **A.** Cells were treated with sorafenib (0-16nM) or quizartinib (0-8nM) either alone or in combination with ATO (0-4μM) for 24 hours. Cell proliferation was measured in quadruplicate by MTT assay. **B.** CFU counts at 9 and 14 days of Molm14 and MV4;11 cells cultured in methylcellulose-based medium (Stemcell Technologies, H4230) treated with sorafenib (4nM) and/or ATO (1μM). Data indicate average ± SD. N=3. Representative images of colonies are shown below. Images were acquired at room temperature using a Nikon Eclipse Ti-E inverted microscope imaging system with a Nikon NIS-Elements AR3.0 software.

To evaluate the effect of combination therapy on cell proliferation, AML cells were treated with increasing concentrations of FLT3 TKI alone (sorafenib, quizartinib or midostaurin), ATO alone, or the combination for 24 hours. A significant inhibition of proliferation in response to the combination therapy was observed in the two FLT3/ITD+ Molm14 and MV4;11 cell lines with combination index (CI) values at ED_50_ below 1.0 for both sorafenib and quizartinib (Figure 2A and Table 1). Midostaurin, a FLT3 TKI with dual inhibitory activity against both FLT3/ITD and FLT3/TKD mutations, showed synergistic effects against Molm14, MV4;11 and HB11;19 cells when combined with ATO. As expected, neither sorafenib nor quizartinib (which are not active against the FLT3/TKD mutation of HB11;19 cells) demonstrate synergy against HB11;19 cells when combined with ATO. No synergy was observed between ATO and the 3 drugs against non-FLT3 mutant cells (Table 1, Supplementary Tables 1, 2, Supplementary Figure 2A-C).

**Table 1:** Combination index (CI) values for FLT3 TKIs in combination with ATO

	Molm14	MV4;11	THP-1	U937
ATO + sorafenib	0.65	0.57	>10	>10
ATO + quizartinib	0.46	0.56	>10	>10
ATO + midostaurin	0.98	0.93	2.74	>10

* Data were collected 24 hours after treatment and CI values calculated at ED_50_.

Furthermore, we assessed the effects of the drugs on the colony formation of the cell lines. While sorafenib, quizartinib or ATO alone led to a substantial reduction in colony numbers in both Molm14 and MV4;11 cells, clonogenicity were further decreased upon combination treatment. The colonies that did grow were noted to have greatly reduced size as well (Figure 2B, Supplementary Figure 2D).

### Combination of ATO and FLT3 TKIs increases cell cycle arrest, apoptosis and differentiation of FLT3 mutant leukemic cells

**Figure 3:**
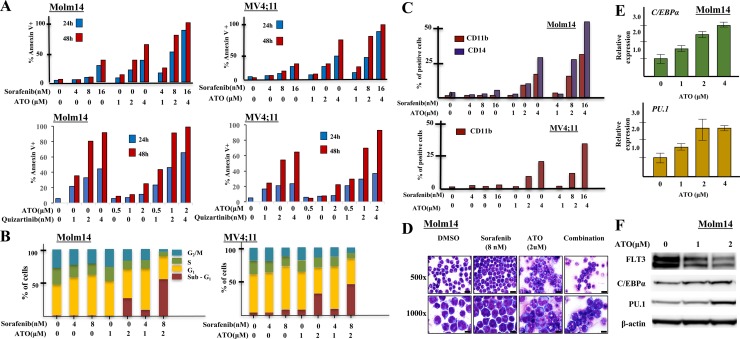
Combining sorafenib with ATO induces strong apoptosis, cell cycle arrest and differentiation in FLT3/ITD+ cell lines **A.** FLT3/ITD+ (Molm14, MV4;11) cells were treated with sorafenib/quizartinib +/− ATO at the indicated concentrations followed by Annexin V binding 24 or 48 hours later. **B.** For cell cycle analysis, cells were stained with propidium iodide (PI) at 48h followed by flow cytometry. **C.** FLT3/ITD+ (Molm14, MV4;11) leukemia cell lines were treated with sorafenib and / or ATO at the indicated concentrations followed by CD11b staining and flow cytometry analysis 48 hours after treatment. **D.** Cellular morphology 48 hours after sorafenib (8nM) and / or ATO (2μM) treatment (500× images, scale bar = 10 microns; 1000× images, scale bar = 20 microns). Wright-Geimsa stained slides were imaged on an Olympus BX46 microscope with an Olympus DP72 camera using Olympus cellSens Standard 1.5 image acquisition software. **E.** C/EBPα and PU.1 expression, determined by Quantitative RT-PCR as normalized to GAPDH, and **F.** Western blotting for C/EBPα and PU.1 expression in Molm14 cells following 48-hour treatment with ATO at the indicated concentrations.

To evaluate the induction of apoptosis in FLT3/ITD+ cells in response to treatment with sorafenib/ATO or quizartinib/ATO, we performed Annexin V and 7-AAD staining on drug-treated Molm14 and MV4;11 cells. Both cells treated with ATO + TKI showed higher levels of apoptosis compared to treatment with either drug alone, in a dose- and time-dependent fashion (Figure 3A). Combination of ATO + midostaurin also induced increased apoptosis in HB11;19 cells (Supplementary Figure 3A). In contrast, in non-FLT3 mutant leukemic cells, ATO resulted in limited induction of apoptosis and no additive effect when combined with FLT3 TKI (Supplementary Figure 3B).

We also examined the influence of sorafenib and ATO on cell cycle kinetics of the FLT3/ITD+ cells. Both sorafenib and ATO induced cell cycle arrest with an increased fraction of cells in G_1_ or sub-G_1_ and a decreased fraction in S and G_2_/M phase. Combination treatment resulted in a significant increase in the fraction of cells in sub-G_1_ (Figure 3B). Little change was noted in the fraction of cells in different stages of the cell cycle in the non-FLT3 mutant cells in response to each drug alone or the combination (Supplementary Figure 3C). These results are consistent with previous reports showing that ATO induces cell cycle arrest in myeloma cells [32, 33].

We next examined whether treatment with the drugs caused any changes in differentiation of the cells. Combination therapy increased CD11b and CD14 expression (markers of myeloid and monocytic differentiation) in the remaining viable Molm14 cells compared to ATO or sorafenib treatment alone. While MV4;11 cells express CD14, expression of CD11b was enhanced by ATO and sorafenib combination treatment (Figure 3C). Morphologically, Molm14 cells treated with 2μM ATO showed evidence of maturation, as characterized by larger cell size, increased cytoplasm with occasional eosinophilic granules, and more complex nuclear location (Figure 3D). THP-1 cells also increased the percentage of cells expressing CD11b in response to ATO, but the addition of sorafenib did not cause any further increase (Supplementary Figure 3D, E).

C/EBPα and PU.1 are two key myeloid regulators known to play pivotal roles in monocytic and granulocytic differentiation [34, 35]. ATO treatment of Molm14 cells increased RNA expression of both of these differentiation regulatory transcription factors (Figure 3E). Western blotting analysis confirmed increased C/EBPα and PU.1 protein levels in the treated cells (Figure 3F).

### ATO decreases FLT3 expression and in combination with FLT3 TKI further inactivates FLT3 and its downstream pathways

**Figure 4:**
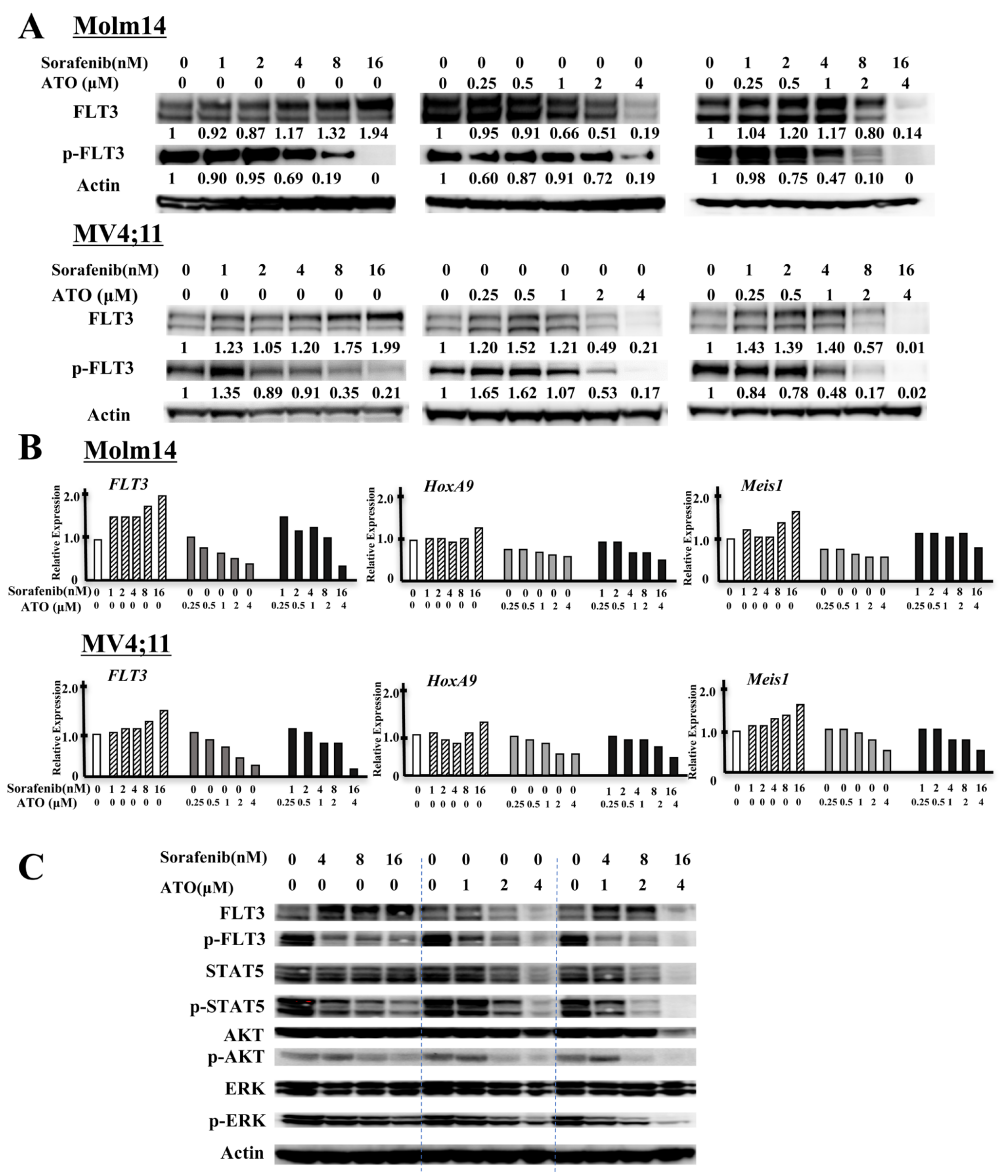
ATO decreases protein levels of FLT3 and shows potent inactivation of FLT3 and its downstream signals in combination with FLT3 TKI **A.** Levels of total FLT3, phospho-FLT3 (pFLT3) in FLT3/ITD+ Molm14 and MV4;11 leukemia cells treated with sorafenib, ATO, or combination for 24 hours. Numbers below pFLT3 and FLT3 are the values of each individual band relative to untreated sample (normalized to actin control). **B.** Quantitative RT-PCR of expression of FLT3, HOXA9 and MEIS1 in FLT3/ITD+ (Molm14, MV4;11) leukemia cell lines treated with sorafenib, ATO, or the combination for 24 hours at the indicated concentration. Values were normalized to GAPDH. **C.** Western blot analysis showing FLT3 and its downstream signaling proteins (STAT5, AKT and ERK) as well as their phosphorylated levels in Molm14 cells treated with sorafenib (4~16nM) and/or ATO (1~4μM) for 24 hours.

We next investigated the mechanism(s) by which ATO treatment affects cell proliferation and differentiation in FLT3/ITD+ cells, and how ATO combines with FLT3 TKI to kill those cells. To that end, we examined expression and activity of FLT3 and its downstream targets in the presence of ATO and FLT3 TKIs in Molm14 and MV4;11 cells. 24 hours after sorafenib or quizartinib treatment, as expected, these cells displayed inhibition of phosphorylated FLT3 in a dose-dependent manner (Figure 4A, Supplementary Figure 4A). As has been previously reported, a significant increase in the level of glycosylated (mature-form, MW 155kDa) but not unglycosylated (immature form, MW 130kDa) FLT3 accumulates once FLT3 kinase activity was inhibited. In contrast, ATO treatment significantly reduced expression of both the mature and immature forms of FLT3. The addition of ATO to sorafenib diminished the extent of increase in FLT3 expression caused by sorafenib treatment alone. The addition of ATO to sorafenib treatment further decreased the level of FLT3 phosphorylation. In FLT3/WT+ THP-1 cells, ATO and the combination decreased protein levels of FLT3 to a lesser extent (Supplementary Figure 4B).

qPCR analysis showed an increase in FLT3 RNA expression in sorafenib-treated Molm14 cells, whereas ATO or ATO + sorafenib significantly inhibited FLT3 RNA expression (Figure 4B). HOXA9 and MEIS1 are known to be major transcriptional regulators of FLT3 expression [36, 37]. qPCR analysis revealed that FLT3 TKI treatment of Molm14 cells increased expression levels of both HOXA9 and MEIS1 RNA. ATO or the combination treatment reduced expression of both genes, correlating with the observed reduced FLT3 expression. ATO also reduced FLT3 expression and its transcriptional regulators in FLT3/WT+ THP-1 cells (Supplementary Figure 4C).

To investigate whether the combination of ATO with FLT3 TKIs affects signaling downstream of FLT3, we analyzed the activity of STAT5, AKT, and ERK in Molm14 cells treated with sorafenib + ATO. ATO + sorafenib further reduced the activation levels of STAT5, AKT, and ERK seen with treatment with sorafenib alone. It is notable that the combination has persistent inhibitory effects on ERK, which was recently shown to adaptively rebound in response to 24 hours of FLT3 inhibition (Figure 4C) [38]. Similar results were found in ATO + quizartinib-treated MV4;11 cells (Supplementary Figure 4A).

### ATO reduces autocrine/paracrine activation by FLT3 ligand through down-regulation of surface FLT3 protein

**Figure 5:**
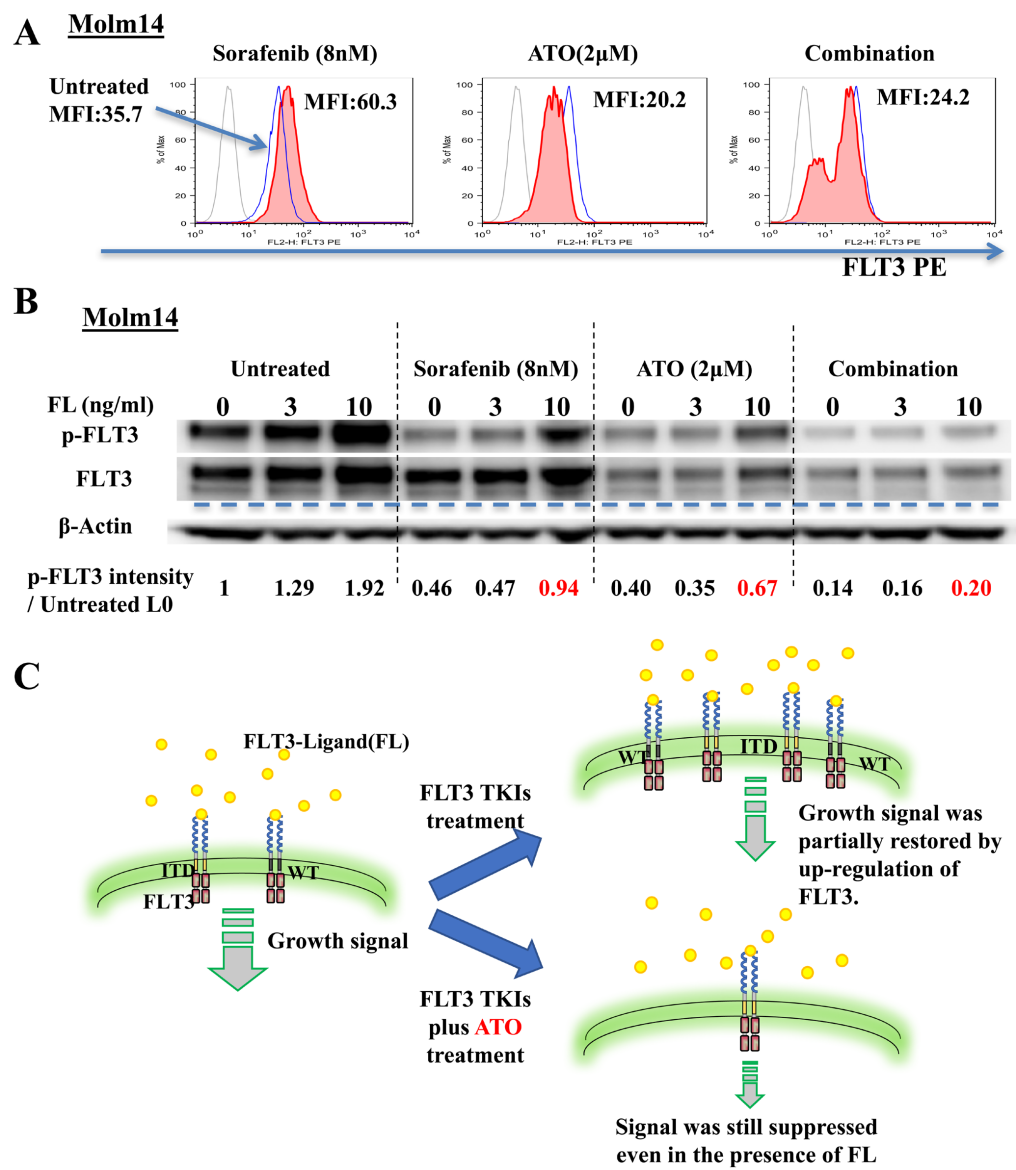
ATO induces down-regulation of surface FLT3 and reduces autocrine/paracrine activation by FLT3-Ligand **A.** Flow cytometry analysis of surface FLT3 expression on Molm14 cells treated with sorafenib (8nM), ATO (2μM), or the combination for 24 hours. Light solid line indicates immunoglobulin isotype. Heavy solid line (blue) indicates Untreated cells. Filled line indicates cells treated as indicated. MFI: Mean Fluorescence Intensity. **B.** Expression and phosphorylation levels of FLT3 in Molm14 cells treated with sorafenib (8nM), ATO (2μM), or the Combination for 24 hours and then stimulated with FLT3-Ligand (0-10ng/ml) for 10 minutes. **C.** Illustration of FLT3 on the surface and growth signaling from it in FLT3/ITD+ cells treated with FLT3 TKIs alone or in combination with ATO.

FLT3 ligand (FL) levels are known to be increased by both FLT3 TKI and chemotherapy treatment of patients with leukemia. This increased level has been shown to increase the level of FLT3 phosphorylation and shifts the dose-response curve to the right (i.e, more resistant) in patients being treated with FLT3 TKI. In order for FL to cause this shift, surface FLT3 must be present in order to bind its ligand [39–41]. To determine the effect of ATO on the surface expression levels of FLT3, we utilized flow cytometry analysis for CD135 (FLT3) expression. Treatment with sorafenib (8nM) increased the Mean Fluorescence Intensity (MFI) of CD135 to nearly twice that of untreated cells whereas ATO (2μM) and combination treatment decreased the MFI (Figure 5A). Next, we evaluated the response to FL of FLT3 phosphorylation under each of these conditions. Enhanced activation/phosphorylation of FLT3 by FL was observed in the untreated Molm14 cells. FL addition restored phosphorylated FLT3 to near baseline levels despite sorafenib treatment. ATO and combination treatment both greatly reduced the FL-stimulated increase of phosphorylation of FLT3 (Figure 5B). The cartoon summarizes these findings. (Figure 5C).

### ATO facilitates poly-ubiquitination and degradation of FLT3 partly through reducing the binding of mutant FLT3 to the deubiquitinating enzyme USP10

**Figure 6:**
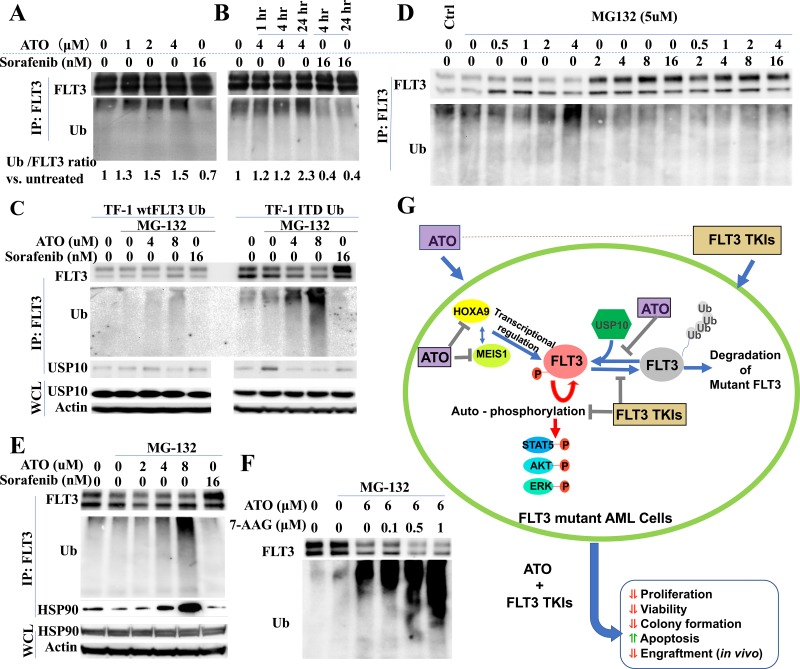
ATO induces poly-ubiquitination and degradation of FLT3 **A.**, **B.** TF-1/ITD-Ub cells were treated with ATO or sorafenib followed by immunoprecipitation with FLT3 antibody and Western blotting. Signal intensity of Ubiquitin relative to FLT3 *vs*. control is shown. **C.**, **D.**, **E.** TF1/WT-Ub or TF1/ITD-Ub cells were treated with 5μM MG-132 for 1 hour followed by 4-hour treatment with ATO or sorafenib. Western blotting was conducted following immunoprecipitation with anti-FLT3 antibodies or using whole cell lysate (WCL). **F.** TF1-ITD-Ub cells were treated with 5μM MG-132 for 1 hour followed by 17-AAG and/or ATO treatment for 4 hours. Cell lysates were immunoprecipitated with anti-FLT3 antibodies followed by Western blotting for FLT3 or Ubiquitin proteins. **G.** Summary of the mechanisms for the synergistic combination of ATO and FLT3 TKIs in FLT3 mutant AML cells.

Several reports have demonstrated that ATO induces poly-ubiquitination and degradation of several mutant oncogenic proteins, including mutated p53 and NPMc [14, 17]. To investigate whether ATO induces ubiquitination and degradation of FLT3/ITD, we transduced a ubiquitin-expressing lentivirus into TF1/FLT3/WT and TF1/FLT3/ITD cells (TF1/wtFLT3-Ub and TF1/ITD-Ub). Co-immunoprecipitation assays demonstrated that treatment of TF1/ITD-Ub cells with ATO resulted in enhanced ubiquitin co-precipitating with FLT3 protein in a time- and dose-dependent manner. In contrast, ubiquitin binding with FLT3 protein was greatly decreased when cells were treated with sorafenib (Figure 6A, 6B).

As ubiquitination triggers the ubiquitin-proteasome pathway leading to a protein degradation cascade, we investigated whether the increased ubiquitination observed in ATO-treated FLT3/ITD+ cells also causes FLT3 degradation. When TF1/wtFLT3-Ub and TF1/ITD-Ub cells were treated with the proteosomal inhibitor MG-132 (to reduce the rate of degradation so the products of partial cleavage can be visualized) a smear towards lower molecular weights can be observed for ubiquitin in the ATO treated lanes in a dose dependent manner. Only a weak ubiquitin signal was detected in TF1/wtFLT3-Ub treated with ATO (Figure 6C). This result shows that ATO preferably induces poly-ubiqutination of mutant FLT3/ITD protein compared to FLT3/WT protein.

Increase in FLT3 expression upon TKI treatment could be caused by increased transcription (as shown in Figure 4B) or reduced degradation, or both. To further elucidate its association with FLT3 ubiquitination and degradation, we performed co-immunoprecipitation assay on sorafenib-treated TF1/ITD-Ub cells. We found that while ATO promoted the ubiquitination of FLT3, sorafenib reduced FLT3 ubiquitination. This occurred in a dose-dependent manner and in line with the observed increased levels of FLT3. The addition of higher concentrations of ATO (>2μM) to the sorafenib-treated cells increased the levels of ubiquitination of FLT3 (Figure 6D, Supplementary Figure 5). Thus, FLT3 TKI treatment decreases ubiquitination and degradation of FLT3 as well as increases its transcription, causing increased levels of FLT3 protein in FLT3 mutant cells. In contrast, ATO treatment reduces FLT3 expression through enhanced ubiquitination and decreased transcription.

USP10 is a deubiquitinating enzyme, which specifically removes ubiquitin from its target substrates, such as p53, thus preventing these proteins from degradation. It was recently reported that inhibition of USP10 also induces degradation of FLT3/ITD [42]. Since ATO treatment induces ubiquitination of FLT3/ITD protein, we investigated whether increased ubiquitination in ATO-treated FLT3/ITD cells is the consequence of the dissociation of USP10 with mutant FLT3 protein in response to ATO-treatment. Co-immunoprecipitation assays showed that ATO treatment led to significantly reduced binding of USP10 with FLT3 protein in TF1/ITD-Ub cells. In contrast, only a weak decrease in USP10 binding was observed in TF1/wtFLT3-Ub cells when treated with a higher concentration of ATO (8μM) (Figure 6C). These results suggest that ATO treatment results in ubiquitination of mutant FLT3 protein, at least partly through the reduced binding with the deubiquitinating enzyme USP10.

Molecular chaperones, including heat shock protein 90 (Hsp90), form complexes and stabilize client proteins [43]. Client proteins not being chaperoned by the mature Hsp90 complex are degraded through the ubiquitin proteasome pathway. Previous reports showed that Hsp90 is overexpressed in tumor cells and protects oncogenic mutant proteins including FLT3/ITD from ubiquitination and degradation [44–46]. Surprisingly, we found increased Hsp90 protein binding with FLT3 in the ATO-treated cells (Figure 6E). Therefore, while ATO facilitate polyubiqutination and degradation of FLT3 protein it also appears to increase binding of Hsp90, which, partially protects FLT3 protein from ubiqutination and degradation. Based on this hypothesis, we added 17-AAG, an Hsp90 inhibitor, into the ATO-treatment group. Co-immunoprecipitation revealed that 17-AAG accelerated ubiqutination and degradation of FLT3/ITD in cells treated with ATO (Figure 6F). Our results suggest that inhibition of Hsp90 may be desirable in FLT3/ITD+ leukemic cells treated with ATO to enhance degradation of the mutant FLT3 protein. The proposed mechanisms for the synergistic effects of ATO and FLT3 TKIs in FLT3 mutant AML cells are summarized in Figure 6G.

### The addition of ATO to TKI treatment of FLT3/ITD+ patient primary leukemic cells further suppresses their growth, viability and clonogenicity

To investigate whether the observed additive/synergistic effects of ATO plus FLT3 TKI observed for FLT3/ITD+ cells is also true for primary FLT3/ITD+ AML cells, we performed a similar series of experiments using patient samples.

**Figure 7:**
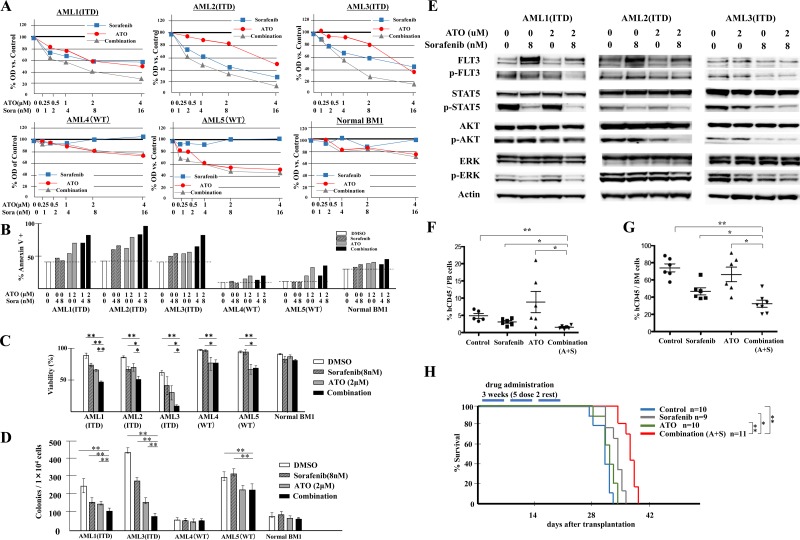
Combination of ATO with sorafenib increases anti-leukemic effects on FLT3/ITD+ patient primary cells and inhibits growth of transplanted leukemia cells *in vivo* BM samples from AML patients with mutant (FLT3/ITD, patients AML1, 2 and 3) or wild-type FLT3 (patients AML4 and 5) or a healthy donor (Normal BM1) were treated with sorafenib and/or ATO. **A.** MTT assay 72 hours after treatment, **B.** Flow-cytometry-based apoptosis assay, **C.** trypan blue staining and **E.** Western blotting analysis were conducted 48 hours after treatment; **D.** CFU-L assay were performed and colonies were counted after 10 days of incubation. **F.**, **G.** Engraftment in peripheral blood and BM of recipients on day 25, represented by percentage of human CD45^+^ cells out of total mononuclear cells. **H.** Kaplan-Meier survival curve for recipients receiving the indicated treatments for 3 weeks (5 times per week) starting on day 3 after transplantation. Error bars indicate average ± SD. **p < 0.001, *p < 0.05.

Samples were treated with sorafenib, ATO or the combination for 72 hours, and analyzed for cell proliferation by MTT assay. The combination shows synergistic anti-proliferative effects on FLT3/ITD AML patient samples AML1, AML2 and AML3, with CI values of 0.658, 0.733 and 0.827, respectively at the ED_50_. In FLT3/WT leukemic samples, variable sensitivity to ATO was observed, but no additive or synergistic effect was seen in response to the combination (Figure 7A). Significant increases in apoptosis and decreases in cell viability were also noted in FLT3/ITD+ leukemia samples in response to combination treatment whereas little change was observed in FLT3/WT leukemic and the normal BM MNC samples (Figure 7B, 7C). We also performed CFU assays on the primary leukemia samples and normal BM MNC and observed significant decreases in the colonies formed in response to the combination *vs*. that of either drug alone only for the FLT3/ITD+ leukemic samples (Figure 7D). The combination of ATO and FLT3 TKI resulted in potent inhibition on downstream targets of FLT3 signaling in FLT3/ITD+ leukemia samples, as was seen previously for the FLT3/ITD+ cell lines (Figure 7E).

### Combination of ATO with sorafenib i*n vivo* modestly reduces leukemic cell engraftment in mice transplanted with FLT3/ITD+ Molm14 cells

To determine whether the combination of ATO plus FLT3 TKI treatment was effective *in vivo*, we injected NSG mice with 0.5×10^6^ Molm14 cells. Three days after transplantation, vehicle alone, sorafenib alone, ATO alone, or the combination of sorafenib plus were administrated for 3 weeks. On day 25 after transplantation, significant reductions were noted in the fraction of leukemic cells (hCD45+) in PB of the sorafenib and combination groups (Figure 7F). ATO treatment appeared to induce mobilization from BM and thus showed an increased fraction of cells in the PB. In the BM, the combination treatment of ATO plus sorafenib resulted in a moderate but statistically significant reduction in leukemic cells compared to either drug alone (Figure 7G).

We also followed the survival of a Molm14-injected cohort of NSG mice treated with the different drug combinations. Only the sorafenib and combination treatment groups showed a modestly prolonged survival compared to the vehicle control group. The combination group showed improved survival compared to the sorafenib alone group (Figure 7H). These results suggest that the combination of ATO with sorafenib modestly reduces the engraftment and expansion of FLT3/ITD+ cells *in vivo.*

## DISCUSSION

Leukemic cells have a number of ways of avoiding cytotoxicity induced by FLT3 TKIs. Increasing expression of FLT3 receptor and its ligand both occur in AML cell lines and primary cells treated with FLT3 TKIs and can at least partially reverse kinase inhibition [40, 41]. Eventually, these leukemic cells can acquire resistance mutations within the TKD or other areas of FLT3 rendering them insensitive to FLT3 TKI. They can also develop mutations or upregulate signaling in other pathways leading to FLT3-independence as a mechanism for secondary drug resistance [47].

In this study, morphologic and phenotypic differentiation was induced in Molm14 cells treated with ATO *via* up-regulation of C/EBPα and PU.1 expression. ATO has previously been observed to induce monocytic and granulocytic differentiation of non-APL AML cell lines and patient primary cells. ATRA plus ATO treatment induces HL-60 differentiation *via* down-regulated expression of proteinase 3 (PRTN3) and up-regulation of PU.1 expression [48]. The combination of ATO and Nilotinib induced macrophage and granulocyte differentiation of primary CML myeloid blast crisis leukemic cells [49]. Thus, the addition of ATO to help induce differentiation of FLT3/ITD+ AML blasts in combination with FLT3-TKIs has analogous precedents.

It has also been shown that compensatory up-regulation of FLT3 expression occurs in leukemic cells treated with FLT3 TKIs. FLT3 ligand (FL) is also up-regulated through feedback loops when FLT3 signaling is inhibited with FLT3 TKI. In leukemia cells, FL stimulates auto/paracrine signaling and impedes the efficacy of FLT3 TKIs [40, 41]. There are several reports that ATO induces activation of ERK1/2 and MAPKα in leukemic cell lines [50, 51]. Reactivation of ERK by 24 hours as an adaptive response to FLT3 TKI inhibition has recently been reported [38]. Interestingly, the combination of ATO and FLT3 TKI showed stable inhibition of ERK activation in Molm14 and patient leukemic cells. The synergistic combination of ATO with FLT3 TKIs likely works in overcoming resistance mechanisms including auto/paracrine FL stimulation, FLT3 overexpression and activation of alternative pathways by inhibition of FLT3 up-regulation. In this report, we show that in contrast, ATO reduces the level of FLT3 protein through enhanced degradation and repressed production, thus preventing leukemic cells from up-regulation of FLT3 in response to TKI treatment. This blocks the ability of FL to stimulate signaling through the receptor and to interfere with the cytotoxic effect of FLT3 TKIs.

Previous work has shown that ATO represses NF-κB activation and induces cell apoptosis in leukemia cells [52]. Our results showed that ATO also reduces expression of HOXA9 and MEIS1. HOXA9 regulates its own expression by a feedback loop requiring binding between HOXA9 and NF-κB. In addition, HOXA9 and MEIS1 are known to be major transcriptional regulators of FLT3 expression [36, 37]. It is thus likely that NF-κB is involved in the ATO-induced down regulation of HOXA9, MEIS1 and FLT3.

FLT3 protein gets degraded through the ubiquitin-proteasome pathway. The ubiquitination process involves the action of ubiquitin-activating (E1), -conjugating (E2) or -ligating (E3) enzymes as well as deubiquitinating enzymes (DUBs) [53]. While phosphorylated wild-type FLT3 and constitutively autophosphorylated FLT3/ITD are poly-ubiquitinated and degraded through E2 ubiquitin conjugase UBCH8 and E3 ubiquitin ligase SIAH1, these enzymes are up-regulated by HDACi [54]. A more recent study revealed that USP10, a deubiquitinating enzyme, is essential for the stabilization of FLT3 protein and inhibition of USP10 induces the degradation of FLT3/ITD, as well as FLT3/TKD, albeit to a lesser extent [42]. This implies that compared to wild-type FLT3, FLT3/ITD and FLT3/TKD mutations may depend more on USP10 for their stabilization, and are thus more susceptible to ubiquitination targeting USP10. The data in our report demonstrated that ATO treatment reduces the binding of USP10 to FLT3 protein, which might partly contribute to the enhanced ubiquitination of mutant FLT3 protein.

Hsp90 is an important chaperone molecule assisting in folding and preventing client proteins from ubiquitination and degradation [44]. FLT3/ITD mutations result in a higher energy of folding and is therefore unstable, making it more dependent on the Hsp90 chaperone in leukemic cells [45, 55]. We found increased HSP90 protein binding with FLT3 in the ATO-treated cells perhaps as a result of its further destabilization by ubiquitination. The addition of the Hsp90 inhibitor 17-AAG facilitated the further ubiquitination and degradation of FLT3 protein induced by ATO. These results suggest that inhibition of Hsp90 may be advantageous in leukemic cells treated with ATO in terms of degradation of oncogenic proteins.

It is notable that in the *in vivo* model, the engraftment levels in all of the four groups were still high and the survival time differences were modest despite statistical significances for both. We believe the modest results from the *in vivo* assays *vs*. the more extensive differences in the *in vitro* assays have to do with the effect of the surrounding stromal cells *in vivo*. The MV4;11 cells growing *in vitro* is highly dependent on constitutively activated FLT3 signaling therefore their sensitivity to FLT3 inhibitors determines their survival/proliferation. When growing *in vivo*, the presence of stromal cells and cytokines provides multiple survival signals making them less dependent on the FLT3 pathway.

In conclusion, this report has demonstrated that ATO exerts synergistic anti-leukemic effects together with FLT3 TKI through down regulation of FLT3 expression. This mechanism can overcome the increased expression of FLT3 with autocrine activation of signaling by FL seen in response to FLT3 TKIs. ATO has the potential to improve the efficacy of FLT3 TKIs in the treatment of FLT3/ITD+ leukemia.

## MATERIALS AND METHODS

### Cell lines, primary cells and reagents

AML cell lines were cultured in RPMI1640 medium supplemented with 10% fetal calf serum (FCS) and 1% penicillin/streptomycin. Primary samples were acquired upon Johns Hopkins institutional review board (IRB) approval with written informed consent from all patients and healthy volunteers in accordance with the Declaration of Helsinki. Bone marrow (BM) mononuclear cells (MNCs) were isolated by centrifugation and stored in liquid nitrogen until use.

Arsenic trioxide (Sigma) was dissolved in 1M NaOH at 60°C for 20 min and prepared as a 25mM stock solution. Quizartinib (AC220), sorafenib and lestaurtinib (LC Laboratories) were dissolved in dimethylsulfoxide (DMSO) and prepared as 10mM stock solutions in RPMI with 0.1% DMSO. Antibodies used are listed in Supplementary Table 3.

### Cell proliferation assay

Cell proliferation was measured using MTT assay as described previously [56]. IC_50_ was calculated by linear regression analysis of optical density.

### Colony formation assay

Colony forming unit (CFU) assays were performed as described previously [56]. Colony numbers were counted after 9-14 days of cell culture.

### Flow cytometry analysis

Flow cytometry analysis was performed using BD FACSCalibur (BD Biosciences) as described previously [56]. All data were analyzed with FlowJo analysis software (Version 9.9.3, Tree Star).

### Quantitative RT-PCR analysis

RNA extraction was performed as previously described and Quantitative RT-PCR was conducted using an CFX96 real-time PCR system (Bio-Rad) [57]. The levels of transcripts were normalized based on that of GAPDH. Primer sequences are shown in Supplementary Table 4.

### Western blotting and immunoprecipitation

Cells were lysed with Cell Lysis Buffer (Cell signaling) and Complete Mini Protease Inhibitor Cocktail (Roche). Immunoprecipitation and Western blotting analysis were performed as described previously [56].

### *In vivo* mouse experiments

All animal procedures were conducted in accordance with the policy of the Johns Hopkins Animal Care and Use Committee. NOD/SCID-IL-2Rγ^−/−^ (NSG) mice were transplanted with MV4;11 cells *via* lateral tail vein injection. 3 days later, mice were treated with indicated drugs in a 5-days-on/2-days-off schedule. In one cohort, mice were euthanized on day 25 for engraftment analysis. In the other, injected mice were monitored for survival.

### Statistical analysis

Statistical analysis was performed with Student's t-test and log-rank test by use of the GraphPad software analysis program (Prism). P values of less than 0.05 were considered to be statistically significant. All data are presented as the mean ± standard deviation (SD).

## SUPPLEMENTARY MATERIALS FIGURES AND TABLES



## References

[1] Döhner H, Estey EH, Amadori S, Appelbaum FR, Büchner T, Burnett AK, Dombret H, Fenaux P, Grimwade D, Larson RA, Lo-Coco F, Naoe T, Niederwieser D, European LeukemiaNet (2010). Diagnosis and management of acute myeloid leukemia in adults: recommendations from an international expert panel, on behalf of the European LeukemiaNet. Blood.

[2] Gilliland DG, Griffin JD (2002). The roles of FLT3 in hematopoiesis and leukemia. Blood.

[3] Levis M, Small D (2003). FLT3: ITDoes matter in leukemia. Leukemia.

[4] Chu SH, Small D (2009). Mechanisms of resistance to FLT3 inhibitors. Drug Resist Updat.

[5] Fischer T, Stone RM, Deangelo DJ, Galinsky I, Estey E, Lanza C, Fox E, Ehninger G, Feldman EJ, Schiller GJ, Klimek VM, Nimer SD, Gilliland DG (2010). Phase IIB trial of oral Midostaurin (PKC412), the FMS-like tyrosine kinase 3 receptor (FLT3) and multi-targeted kinase inhibitor, in patients with acute myeloid leukemia and high-risk myelodysplastic syndrome with either wild-type or mutated FLT3. J Clin Oncol.

[6] Knapper S, Burnett AK, Littlewood T, Kell WJ, Agrawal S, Chopra R, Clark R, Levis MJ, Small D (2006). A phase 2 trial of the FLT3 inhibitor lestaurtinib (CEP701) as first-line treatment for older patients with acute myeloid leukemia not considered fit for intensive chemotherapy. Blood.

[7] Man CH, Fung TK, Ho C, Han HH, Chow HC, Ma AC, Choi WW, Lok S, Cheung AM, Eaves C, Kwong YL, Leung AY (2012). Sorafenib treatment of FLT3-ITD(+) acute myeloid leukemia: favorable initial outcome and mechanisms of subsequent nonresponsiveness associated with the emergence of a D835 mutation. Blood.

[8] Smith CC, Wang Q, Chin CS, Salerno S, Damon LE, Levis MJ, Perl AE, Travers KJ, Wang S, Hunt JP, Zarrinkar PP, Schadt EE, Kasarskis A (2012). Validation of ITD mutations in FLT3 as a therapeutic target in human acute myeloid leukaemia. Nature.

[9] Jeanne M, Lallemand-Breitenbach V, Ferhi O, Koken M, Le Bras M, Duffort S, Peres L, Berthier C, Soilihi H, Raught B, de Thé H (2010). PML/RARA oxidation and arsenic binding initiate the antileukemia response of As2O3. Cancer Cell.

[10] Lo-Coco F, Avvisati G, Vignetti M, Thiede C, Orlando SM, Iacobelli S, Ferrara F, Fazi P, Cicconi L, Di Bona E, Specchia G, Sica S, Divona M, Gruppo Italiano Malattie Ematologiche dell'Adulto, German-Austrian Acute Myeloid Leukemia Study Group, Study Alliance Leukemia (2013). Retinoic acid and arsenic trioxide for acute promyelocytic leukemia. N Engl J Med.

[11] Maroui MA, Kheddache-Atmane S, El Asmi F, Dianoux L, Aubry M, Chelbi-Alix MK (2012). Requirement of PML SUMO interacting motif for RNF4- or arsenic trioxide-induced degradation of nuclear PML isoforms. PLoS One.

[12] Zhang XW, Yan XJ, Zhou ZR, Yang FF, Wu ZY, Sun HB, Liang WX, Song AX, Lallemand-Breitenbach V, Jeanne M, Zhang QY, Yang HY, Huang QH (2010). Arsenic trioxide controls the fate of the PML-RARalpha oncoprotein by directly binding PML. Science.

[13] Martelli MP, Gionfriddo I, Mezzasoma F, Milano F, Pierangeli S, Mulas F, Pacini R, Tabarrini A, Pettirossi V, Rossi R, Vetro C, Brunetti L, Sportoletti P (2015). Arsenic trioxide and all-trans retinoic acid target NPM1 mutant oncoprotein levels and induce apoptosis in NPM1-mutated AML cells. Blood.

[14] El Hajj H, Dassouki Z, Berthier C, Raffoux E, Ades L, Legrand O, Hleihel R, Sahin U, Tawil N, Salameh A, Zibara K, Darwiche N, Mohty M (2015). Retinoic acid and arsenic trioxide trigger degradation of mutated NPM1, resulting in apoptosis of AML cells. Blood.

[15] Goussetis DJ, Gounaris E, Wu EJ, Vakana E, Sharma B, Bogyo M, Altman JK, Platanias LC (2012). Autophagic degradation of the BCR-ABL oncoprotein and generation of antileukemic responses by arsenic trioxide. Blood.

[16] Perkins C, Kim CN, Fang G, Bhalla KN (2000). Arsenic induces apoptosis of multidrug-resistant human myeloid leukemia cells that express Bcr-Abl or overexpress MDR, MRP, Bcl-2, or Bcl-x(L). Blood.

[17] Yan W, Jung YS, Zhang Y, Chen X (2014). Arsenic trioxide reactivates proteasome-dependent degradation of mutant p53 protein in cancer cells in part via enhanced expression of Pirh2 E3 ligase. PLoS One.

[18] Bhattacharjee H, Li J, Ksenzenko MY, Rosen BP (1995). Role of cysteinyl residues in metalloactivation of the oxyanion-translocating ArsA ATPase. J Biol Chem.

[19] Cavigelli M, Li WW, Lin A, Su B, Yoshioka K, Karin M (1996). The tumor promoter arsenite stimulates AP-1 activity by inhibiting a JNK phosphatase. EMBO J.

[20] Chen YC, Lin-Shiau SY, Lin JK (1998). Involvement of reactive oxygen species and caspase 3 activation in arsenite-induced apoptosis. J Cell Physiol.

[21] Kapahi P, Takahashi T, Natoli G, Adams SR, Chen Y, Tsien RY, Karin M (2000). Inhibition of NF-kappa B activation by arsenite through reaction with a critical cysteine in the activation loop of Ikappa B kinase. J Biol Chem.

[22] Porter AC, Fanger GR, Vaillancourt RR (1999). Signal transduction pathways regulated by arsenate and arsenite. Oncogene.

[23] Cui X, Kobayashi Y, Akashi M, Okayasu R (2008). Metabolism and the paradoxical effects of arsenic: carcinogenesis and anticancer. Curr Med Chem.

[24] Subbarayan PR, Ardalan B (2014). In the war against solid tumors arsenic trioxide needs partners. J Gastrointest Cancer.

[25] Parmar S, Rundhaugen LM, Boehlke L, Riley M, Nabhan C, Raji A, Frater JL, Tallman MS (2004). Phase II trial of arsenic trioxide in relapsed and refractory acute myeloid leukemia, secondary leukemia and/or newly diagnosed patients at least 65 years old. Leuk Res.

[26] Schiller GJ, Slack J, Hainsworth JD, Mason J, Saleh M, Rizzieri D, Douer D, List AF (2006). Phase II multicenter study of arsenic trioxide in patients with myelodysplastic syndromes. J Clin Oncol.

[27] Vey N, Bosly A, Guerci A, Feremans W, Dombret H, Dreyfus F, Bowen D, Burnett A, Dennis M, Ribrag V, Casadevall N, Legros L, Fenaux P (2006). Arsenic trioxide in patients with myelodysplastic syndromes: a phase II multicenter study. J Clin Oncol.

[28] Evens AM, Tallman MS, Gartenhaus RB (2004). The potential of arsenic trioxide in the treatment of malignant disease: past, present, and future. Leuk Res.

[29] Takahashi S, Harigae H, Yokoyama H, Ishikawa I, Abe S, Imaizumi M, Sasaki T, Kaku M (2006). Synergistic effect of arsenic trioxide and flt3 inhibition on cells with flt3 internal tandem duplication. Int J Hematol.

[30] Wang LN, Tang YL, Zhang YC, Zhang ZH, Liu XJ, Ke ZY, Li Y, Tan HZ, Huang LB, Luo XQ (2017). Arsenic trioxide and all-trans-retinoic acid selectively exert synergistic cytotoxicity against FLT3-ITD AML cells via co-inhibition of FLT3 signaling pathways. Leuk Lymphoma.

[31] Wang R, Li Y, Gong P, Gabrilove J, Waxman S, Jing Y (2018). Arsenic trioxide and sorafenib induce synthetic lethality of FLT3-ITD acute myeloid leukemia cells. Mol Cancer Ther.

[32] Liu Q, Hilsenbeck S, Gazitt Y (2003). Arsenic trioxide-induced apoptosis in myeloma cells: p53-dependent G1 or G2/M cell cycle arrest, activation of caspase-8 or caspase-9, and synergy with APO2/TRAIL. Blood.

[33] Park WH, Seol JG, Kim ES, Hyun JM, Jung CW, Lee CC, Kim BK, Lee YY (2000). Arsenic trioxide-mediated growth inhibition in MC/CAR myeloma cells via cell cycle arrest in association with induction of cyclin-dependent kinase inhibitor, p21, and apoptosis. Cancer Res.

[34] Chen HM, Zhang P, Voso MT, Hohaus S, Gonzalez DA, Glass CK, Zhang DE, Tenen DG (1995). Neutrophils and monocytes express high levels of PU.1 (Spi-1) but not Spi-B. Blood.

[35] Zhang DE, Zhang P, Wang ND, Hetherington CJ, Darlington GJ, Tenen DG (1997). Absence of granulocyte colony-stimulating factor signaling and neutrophil development in CCAAT enhancer binding protein alpha-deficient mice. Proc Natl Acad Sci U S A.

[36] Gwin K, Frank E, Bossou A, Medina KL (2010). Hoxa9 regulates Flt3 in lymphohematopoietic progenitors. J Immunol.

[37] Wang GG, Pasillas MP, Kamps MP (2006). Persistent transactivation by meis1 replaces hox function in myeloid leukemogenesis models: evidence for co-occupancy of meis1-pbx and hox-pbx complexes on promoters of leukemia-associated genes. Mol Cell Biol.

[38] Bruner JK, Ma HS, Li L, Qin AC, Rudek MA, Jones RJ, Levis MJ, Pratz KW, Pratilas CA, Small D (2017). Adaptation to TKI Treatment Reactivates ERK Signaling in Tyrosine Kinase-Driven Leukemias and Other Malignancies. Cancer Res.

[39] Sato T, Yang X, Knapper S, White P, Smith BD, Galkin S, Small D, Burnett A, Levis M (2011). FLT3 ligand impedes the efficacy of FLT3 inhibitors in vitro and in vivo. Blood.

[40] Zheng R, Bailey E, Nguyen B, Yang X, Piloto O, Levis M, Small D (2011). Further activation of FLT3 mutants by FLT3 ligand. Oncogene.

[41] Zheng R, Levis M, Piloto O, Brown P, Baldwin BR, Gorin NC, Beran M, Zhu Z, Ludwig D, Hicklin D, Witte L, Li Y, Small D (2004). FLT3 ligand causes autocrine signaling in acute myeloid leukemia cells. Blood.

[42] Weisberg EL, Schauer NJ, Yang J, Lamberto I, Doherty L, Bhatt S, Nonami A, Meng C, Letai A, Wright R, Tiv H, Gokhale PC, Ritorto MS (2017). Inhibition of USP10 induces degradation of oncogenic FLT3. Nat Chem Biol.

[43] Young JC, Moarefi I, Hartl FU (2001). Hsp90: a specialized but essential protein-folding tool. J Cell Biol.

[44] Isaacs JS, Xu W, Neckers L (2003). Heat shock protein 90 as a molecular target for cancer therapeutics. Cancer Cell.

[45] Minami Y, Kiyoi H, Yamamoto Y, Yamamoto K, Ueda R, Saito H, Naoe T (2002). Selective apoptosis of tandemly duplicated FLT3-transformed leukemia cells by Hsp90 inhibitors. Leukemia.

[46] Oshikawa G, Nagao T, Wu N, Kurosu T, Miura O (2011). c-Cbl and Cbl-b ligases mediate 17-allylaminodemethoxygeldanamycin-induced degradation of autophosphorylated Flt3 kinase with internal tandem duplication through the ubiquitin proteasome pathway. J Biol Chem.

[47] Kindler T, Lipka DB, Fischer T (2010). FLT3 as a therapeutic target in AML: still challenging after all these years. Blood.

[48] Sumi D, Suzukawa K, Himeno S (2016). Arsenic trioxide augments all-trans retinoic acid-induced differentiation of HL-60 cells. Life Sci.

[49] Wang W, Lv FF, Du Y, Li N, Chen Y, Chen L (2015). The effect of nilotinib plus arsenic trioxide on the proliferation and differentiation of primary leukemic cells from patients with chronic myoloid leukemia in blast crisis. Cancer Cell Int.

[50] Beauchamp EM, Kosciuczuk EM, Serrano R, Nanavati D, Swindell EP, Viollet B, O'Halloran TV, Altman JK, Platanias LC (2015). Direct binding of arsenic trioxide to AMPK and generation of inhibitory effects on acute myeloid leukemia precursors. Mol Cancer Ther.

[51] Giafis N, Katsoulidis E, Sassano A, Tallman MS, Higgins LS, Nebreda AR, Davis RJ, Platanias LC (2006). Role of the p38 mitogen-activated protein kinase pathway in the generation of arsenic trioxide-dependent cellular responses. Cancer Res.

[52] Wetzler M, Brady MT, Tracy E, Li ZR, Donohue KA, O'Loughlin KL, Cheng Y, Mortazavi A, McDonald AA, Kunapuli P, Wallace PK, Baer MR, Cowell JK, Baumann H (2006). Arsenic trioxide affects signal transducer and activator of transcription proteins through alteration of protein tyrosine kinase phosphorylation. Clin Cancer Res.

[53] Komander D, Rape M (2012). The ubiquitin code. Annu Rev Biochem.

[54] Buchwald M, Pietschmann K, Müller JP, Böhmer FD, Heinzel T, Krämer OH (2010). Ubiquitin conjugase UBCH8 targets active FMS-like tyrosine kinase 3 for proteasomal degradation. Leukemia.

[55] Yu C, Kancha RK, Duyster J (2014). Targeting oncoprotein stability overcomes drug resistance caused by FLT3 kinase domain mutations. PLoS One.

[56] Ma HS, Greenblatt SM, Shirley CM, Duffield AS, Bruner JK, Li L, Nguyen B, Jung E, Aplan PD, Ghiaur G, Jones RJ, Small D (2016). All-trans retinoic acid synergizes with FLT3 inhibition to eliminate FLT3/ITD+ leukemia stem cells in vitro and in vivo. Blood.

[57] Kim KT, Baird K, Ahn JY, Meltzer P, Lilly M, Levis M, Small D (2005). Pim-1 is up-regulated by constitutively activated FLT3 and plays a role in FLT3-mediated cell survival. Blood.

