# Supplementary material for: Combination of ATO with FLT3 TKIs eliminates FLT3/ITD+ leukemia cells through reduced expression of FLT3
Source: Oncotarget. 2018 Aug 31;9(68):32885–99. doi: 10.18632/oncotarget.25972 (PMC6152471; doi:10.18632/oncotarget.25972)
Supplement: Supplementary file 1 [file oncotarget-09-32885-s001.pdf]

High degree of intratumor single-gene polyclonality of oncogenic activated BRAF kinase in malignant melanoma

SUPPLEMENTARY MATERIALS

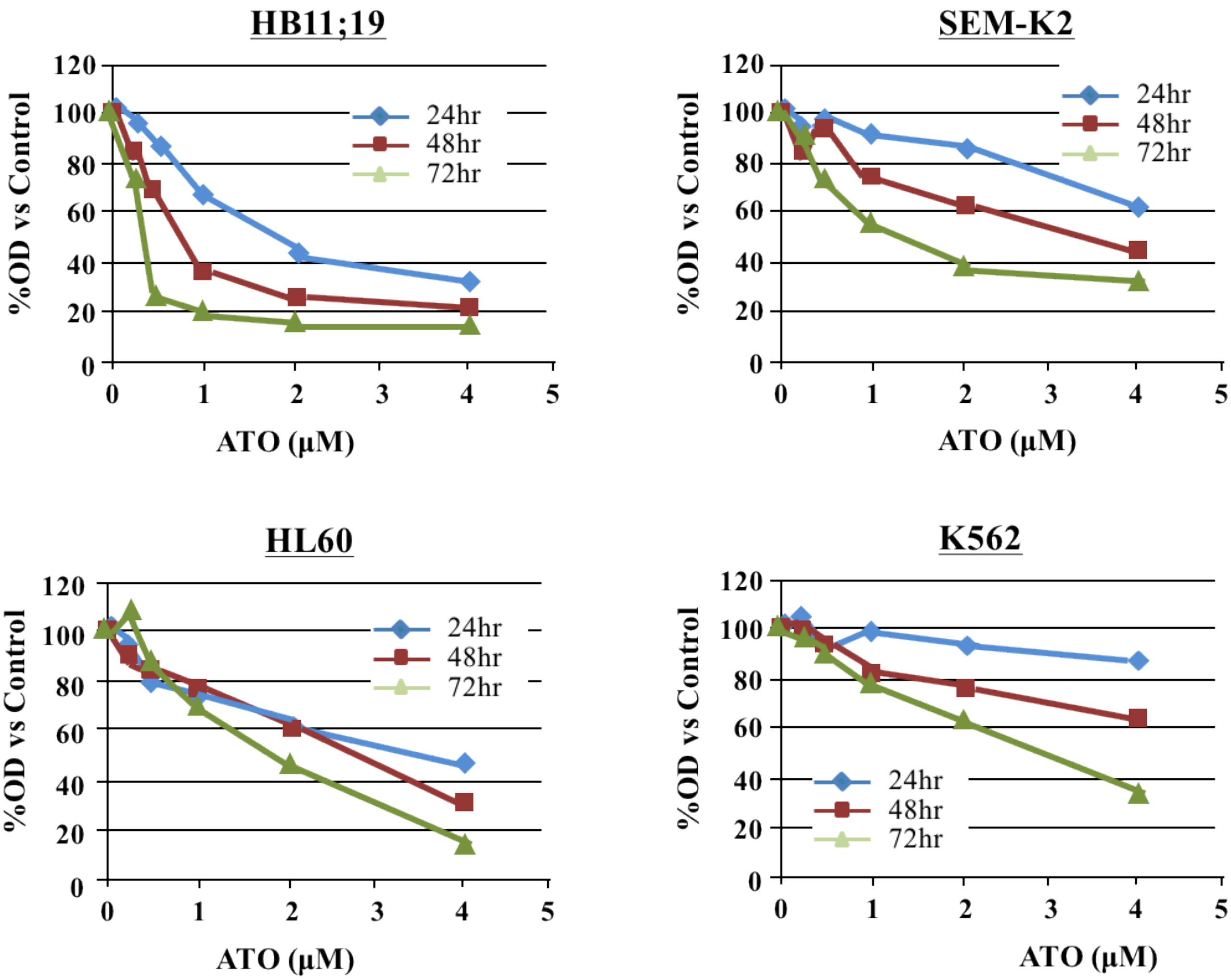

Supplementary Figure 1: FLT3 mutant cells are more sensitive to ATO treatment compared with FLT3/WT leukemic cells or cells not expressing FLT3. FLT3/1TD+ cell lines (HB11;19), FLT3/WT (SEM-K2)or cells not expressing FLT3 (HL 60 and K562) were treated with ATO for up to 72 hours. Cells were subjected to MTT-based cell proliferation assays.

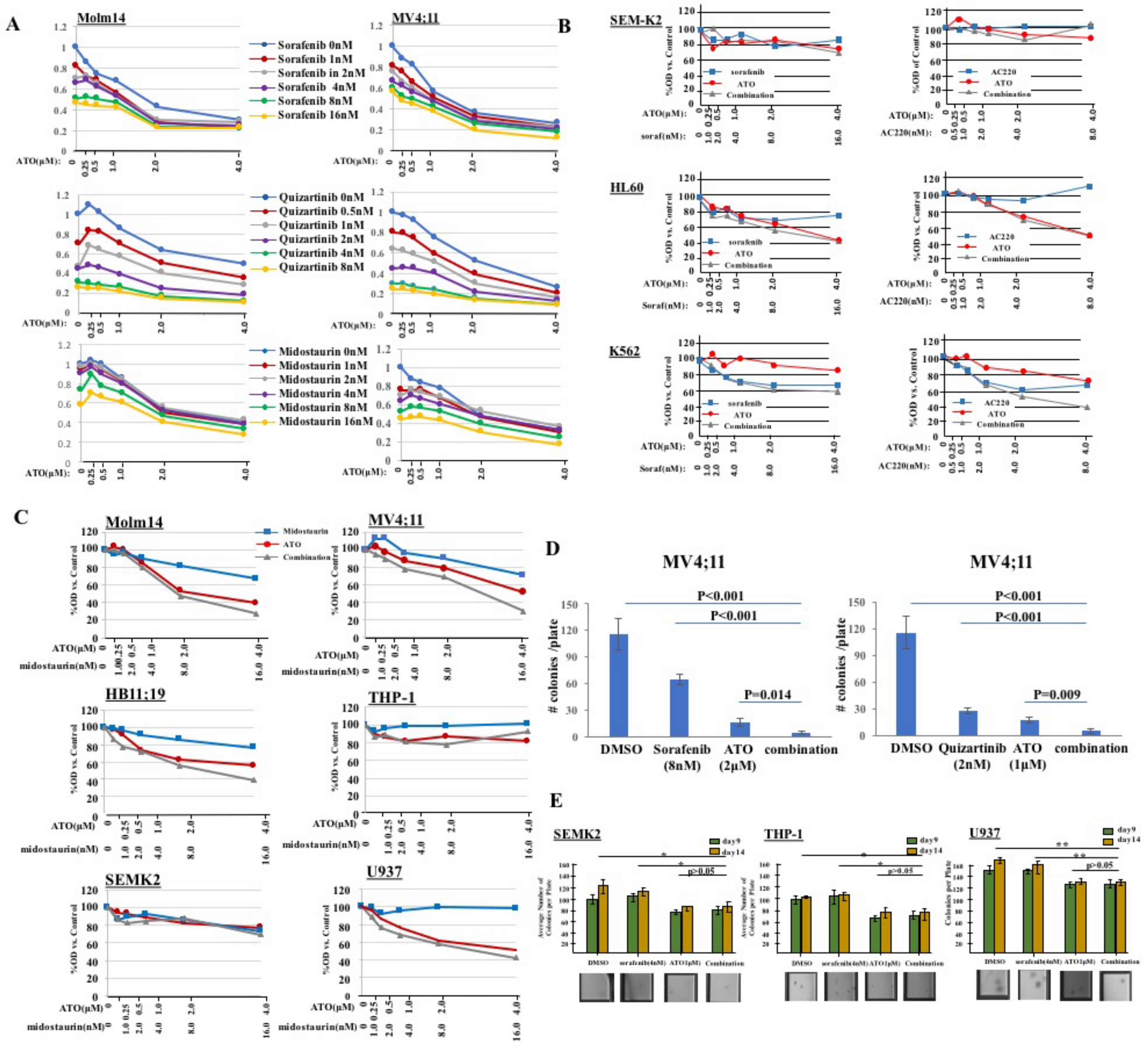

Supplementary Figure 2: Combination of ATO and FLT3 TKIs show synergistic effect on the proliferation and clonogenicity of FLT3/ITD mutant but not non-FLT3 mutant cell lines. (**A-C**) Cells were treated with sorafenib, quizartinib or midostaurin either alone or in combination with ATO for 24 hours. Cells were subjected to MTT-based cell proliferation assays. (**D**, **E**) CFU counts at 9 (for **D** and **E**) and 14 days (for **E**) of cells cultured in methylcellulose-based medium (Stemcell Technologies, H4230) treated with sorafenib +/- ATO or quizartinib +/- ATO. Data indicate average± SD. $N = 3$. *$p < 0.05$ **$p < 0.01$.

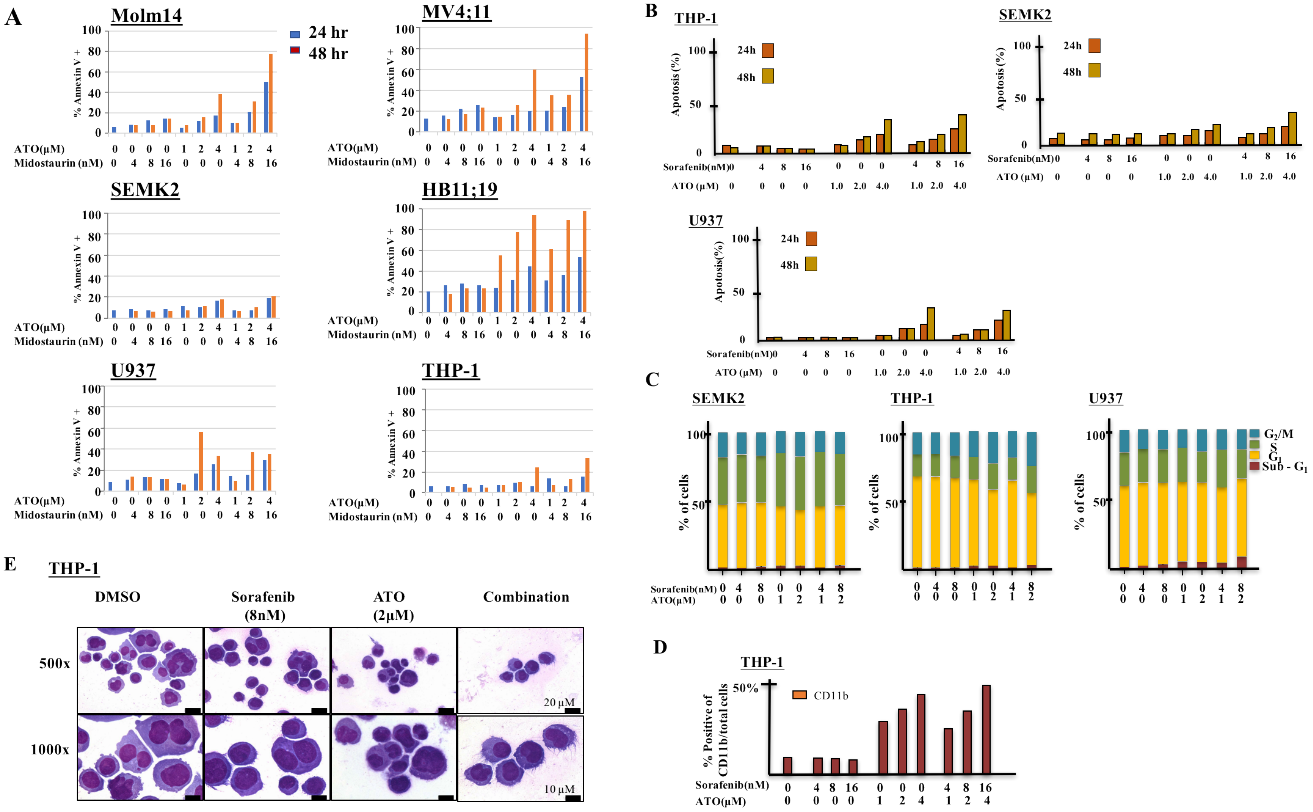

Supplementary Figure 3: Combination of ATO and FLT3 TKIs show synergistic effect on the apoptosis, cell cycling and differentiation of FLT3 mutant but not non FLT3 mutant cell lines. (**A-D**). Cells were treated with sorafenib or midostaurin ATO for 24 or 48 hours and subjected to Annexin V binding assay, cell cycle analysis or CD11b staining. Data indicate average ± SD. *N* = 3. (**E**) Cellular morphology 48 hours after sorefenib (8nM) and I or ATO (2µM) treatment (500x images, scale bar= 10 microns; 1000x images, scale bar= 20 microns). Wright-Geimsa stained slides were imaged on an Olympus BX46 microscope with an Olympus DP72 camera using Olympus cellSens Standard 1.5 image acquisition software.

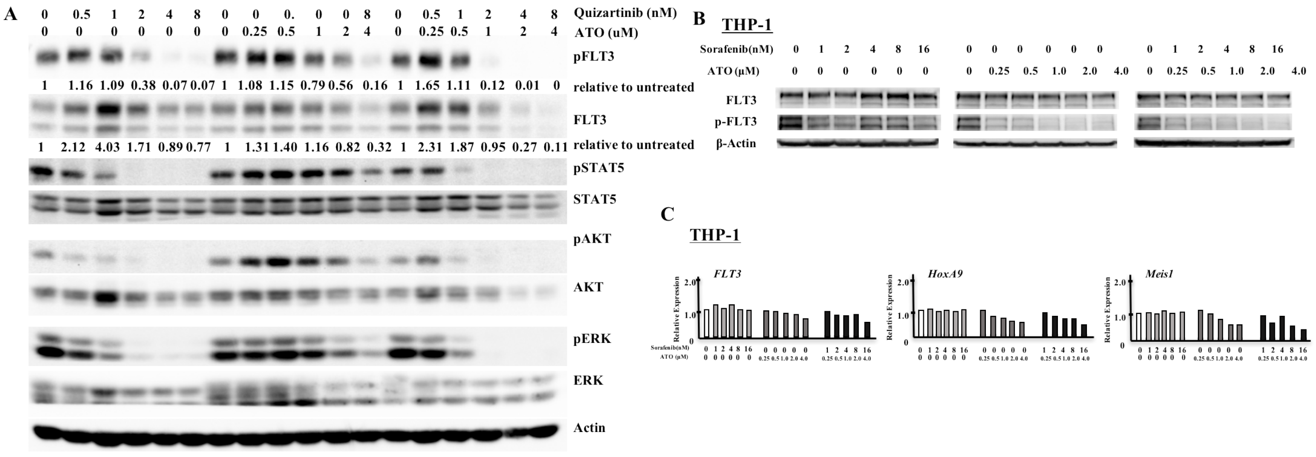

Supplementary Figure 4: FLT3 TKIs synergizes with ATO to decrease the protein level of FLT3 and its downstream signals in FLT3 mutant but not non-FLT3 mutant cells. (**A**) MV4;11 eclls were treated with quizartinib or ATO for 24 hours and subjected to Western blotting analysis. (**B**) Levels of total FLT3 and phospho-FLT3 (pFLT3) in THP-1 cells treated with sorafenib, ATO, or combination for 24 hours. (**C**) Quantitative RT-PCR expression levels of FLT3 and its transcriptional regulators (HoxA9 and Meis1) in THP-1 cells.

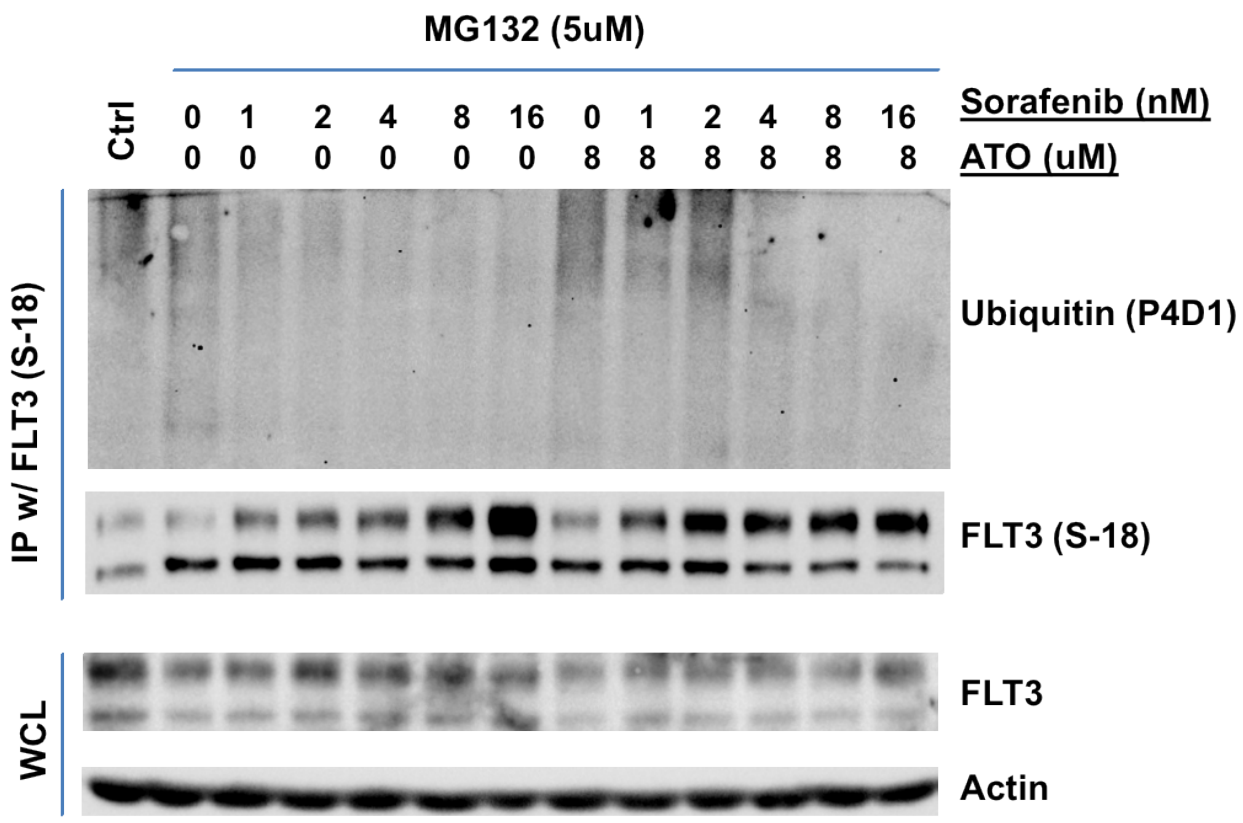

Supplementary Figure 5: Sorafenib inhibits while ATO promotes the poly-Ubiquitination and degradation of FLT3. TF1/ITD-Ub cells were treated with 5 μM MG-132 for 1 hour followed by 4-hour treatment with sorafenib +/- ATO. Western blotting was conducted following immunoprecipitation with anti-FLT3 antibodies or using whole cell lysate (WCL).

Supplementary Table 1: CI values with ATO (at ED_{50}) and Proliferation IC_{50} (nM) values for FLT3 TKI in FLT3 mutant cell lines

		Molm14			MV4;11			HB11;19	
	CI	IC_{50}		CI	IC_{50}		CI	IC_{50}	
		- ATO	+ ATO		- ATO	+ ATO		- ATO	+ ATO
ATO + sorafenib	0.65	21.06	5.31	0.57	19.67	2.75	>10	>100	--
ATO + quizartinib	0.46	2.83	1.00	0.56	2.38	0.97	>10	>100	--
ATO + midostaurin	0.98	24.89	8.59	0.93	15.43	8.37	0.94	39.35	11.57

* Data were collected and analyzed based on MTT assay results 24 hours after TKI treatment.

Supplementary Table 2: Combination index (CI) values at ED_{50} for FLT3 TKIs and ATO

	HB11;19	SEMK2	HL60	K562
ATO + sorafenib	>10	>10	0.86	1.18
ATO + quizartinib	>10	5.81	1.24	0.99
ATO + midostaurin	0.94	>10	--	--

Supplementary Table 3: For the staining of cellular markers were all from BD Biosciences

Antibody	conjugated fluorophore
CD11b	FITC
CD14	FITC
Annexin V	PE
7-amino-actinomycin (7-AAD)	
human CD45	PE
mouse CD45	PerCP-CY5.5

Supplementary Table 4: Primer sequences used for gene expression analysis

Primer	Sequence (5'---3')
FLT3 F	AGGATCAGGTGCTTTTGGAA
FLT3 R	TGCCTCTCTTTCAGAGCTGTC
HoxA9 F	GCGCCTTCTCTGAAAACAAT
HoxA9 R	GTATAGGGGCACCGCTTTTT
Meis1 F	AAATGCCTATCGATTTGGTGA
Meis1 R	AACGAGTAGATGCCGTGTCA
C/EBPα F	TGGACAAGAACAGCAACGAG
C/EBPα R	TTGTCACTGGTCAGCTCCAG
PU.1 F	GAAGACCTGGTGCCCTATGA
PU.1 R	GGGGTGGAAGTCCCAGTAAT
GAPDH F	GAAGGTGAAGGTCGGAGTCA
GAPDH R	AATGAAGGGGTCATTGATGG
